# Alleviating toxic α-Synuclein accumulation by membrane depolarization: evidence from an in vitro model of Parkinson’s disease

**DOI:** 10.1186/s13041-020-00648-8

**Published:** 2020-07-31

**Authors:** Alysia Ross, Viktoria Xing, Ting Ting Wang, Samantha C. Bureau, Giovana A. Link, Teresa Fortin, Hui Zhang, Shawn Hayley, Hongyu Sun

**Affiliations:** 1 Department of Neuroscience, Carleton University, Ottawa, 1125 Colonel By Drive, Ottawa, ON K1S 5B6 Canada; 2 Department of Cellular and Molecular Medicine, University of Ottawa, Ottawa, ON Canada; 3 Department of Neurology, SUNY Downstate Medical center, Brooklyn, NY 11226 USA

**Keywords:** Parkinson’s disease, Membrane depolarization, Preformed fibrils, GABA_A_ receptor, α-Synuclein, Calcium channel, Direct current stimulation

## Abstract

Parkinson’s disease (PD) is characterized by the formation of toxic, fibrillar form alpha-synuclein (α-Syn) protein aggregates in dopaminergic neurons. Accumulating evidence has shown a multifactorial interplay between the intracellular calcium elevation and α-Syn dynamics. However, whether membrane depolarization regulates toxic α-Syn aggregates remains unclear. To understand this better, we used an in vitro α-Syn preformed fibrils (PFF) model of PD in human neural cells. We demonstrated functional membrane depolarization in differentiated SH-SY5Y cells induced by two independent treatments: high extracellular K^+^ and the GABA_A_ receptor blocker picrotoxin. We then observed that these treatments significantly alleviated toxic α-Syn aggregation in PFF-treated SH-SY5Y cells. Moreover, clinically relevant direct current stimulation (DCS) also remarkably decreased toxic α-Syn aggregation in PFF-treated SH-SY5Y cells. Taken together, our findings suggest that membrane depolarization plays an important role in alleviating PFF-induced toxic α-Syn aggregates, and that it may represent a novel therapeutic mechanism for PD.

## Introduction

Parkinson’s disease (PD) is characterized by the formation of insoluble toxic alpha-synuclein (α-Syn) protein aggregates called amyloid fibrils in dopaminergic neurons [18, 19, 21, 38, 54]. α-Syn is a small soluble protein that concentrates at presynaptic terminals throughout the brain [8, 51]. Under physiological conditions, α-Syn monomers facilitate neurotransmitter release through modulating synaptic vesicle maturation and release [6, 19, 44]. However, these soluble α-Syn monomers are prone to undergoing posttranslational modifications under pathological conditions and consequently, form insoluble toxic α-Syn oligomers and fibrils [37]. Toxic α-Syn fibrils aggregate into Lewy bodies and Lewy neurites which represent the key trigger for the onset of synapse deconstruction and subsequent neurodegeneration [5, 18, 43]. Understandably, targeting the mechanisms regulating and clearing toxic α-Syn aggregation has represented a promising disease-modifying strategy for PD treatment [23, 38, 51].

Previous studies using in vivo animal models and in vitro cell culture systems suggest an important role of depolarization-induced disruption of calcium homeostasis in α-Syn monomer secretion and aggregation [25]. In fact, even transient elevation of intracellular calcium by increasing neuronal activities in vivo [52] and in vitro [17, 35] can stimulate the secretion of physiological α-Syn monomers in neurons. On the other hand, in the context of cells overexpressing α-Syn, membrane depolarization leads to an increase in α-Syn aggregation [15, 34]. Thus, it seems that a multifactorial interplay occurs between the intracellular calcium levels and α-Syn dynamics. However, how intracellular toxic α-Syn fibril accumulation is affected by membrane depolarization or the disruption of calcium homeostasis under pathological conditions is completely unknown.

With aggregation, α-Syn fibrils can have toxic consequences, leading to neuronal loss and characteristic PD pathology [9, 26, 30, 51]. To better understand the relationship between membrane depolarization and pathological α-Syn accumulation, we have used an in vitro α-Syn Preformed fibrils (PFF) model of PD in human neural cells. We first assessed whether functional membrane depolarization, induced by treatment of high extracellular K^+^ or GABA_A_ receptor blocker, would alleviate aggregation and promote clearance of toxic α-Syn in PFF-treated SH-SY5Y cells. Secondly, we determined whether clinically relevant direct current stimulation (DCS) could decrease α-Syn aggregation in PFF-treated SH-SY5Y cells. Our findings indicate that these depolarizing treatments did indeed prevent α-Syn pathological aggregation through promoting α-Syn secretion into extracellular medium. This points to the fundamental importance of neuronal activity state in the processing of α-Syn and hence, could lead to the development of novel clinical strategies for PD.

## Methods

### Cell culture

SH-SY5Y cells, a human neuroblastoma cell line, were maintained in Dulbecco’s Modified Eagle Media (DMEM) supplemented with 10% fetal bovine serum (FBS) and 1% penicillin/streptomycin (pen-strep) at 37^o^ in a humidified 90% air and 5% CO_2_ incubator. Cells were plated at a density of 1 × 10^5^ cells/well in cell media, in either flat-bottomed 96 well plates (100 μL/well) for immunohistochemistry, or 24-well plates with coverslips (500 μL/well) for electrophysiology and live-cell imaging. 24-well plates were coverslipped and coated with Poly-D-Lysine (PDL) and Laminin (LM) at 1:1000 in DMEM for 2 h in incubator before plating. Cells were cultured for 2 weeks in complete media (DMEM supplemented with 10% FBS and 1% pen-strep) (week 1) or reduced media (DMEM supplemented with 1% FBS and 1% pen-strep) (week 2) supplemented with 0.1% retinoic acid (RA). Half-media changes were performed every 2 days.

### Whole-cell patch clamp recordings

Differentiated SH-SY5Y cells were collected from 24-well culture plates at 2–3 weeks in culture. Before cells were transferred to the recording chamber under an upright Nikon Eclipse FN1 microscope, culture medium was gradually changed to oxygenated artificial cerebral spinal fluid (ACSF) containing (mM): 124 NaCl, 5 KCl, 1.25 NaH_2_PO_4_, 1.2 MgSO_4_, 26 NaHCO_3_, 2 CaCl_2_, and 10 glucose, in 30 mins at room temperature. Whole-cell patch clamp recordings were obtained from differentiated neurons at 2–3 weeks following the initiation of the differentiation procedure [46]. Patch electrodes with a resistance of 5–10 MΩ were prepared from borosilicate glass capillaries with a Narishige micropipette puller (Model PC-100, Tokyo, Japan). Pipette intracellular solution contains (mM): 130 K-Gluconate, 2 MgCl_2_, 0.6 EGTA, 10 HEPES, 5 KCl, 2 ATP-Mg(Na_2_), pH 7.3. Data were collected using MultiClamp 700B amplifier. To evoke action potentials in these differentiated SH-SY5Y cells, depolarizing rectangular pulses of 500 ms duration (10pA/step) were applied. Signals were filtered at 2 kHz, digitized at 20 kHz by a Digidata 1500 interface, acquired by the pClamp 10.7 software, and analyzed with the Clampfit 10.7 program (Molecular Devices).

### Live cell voltage sensitive dye imaging

As mentioned above, culture medium was gradually changed to oxygenated ACSF containing (mM): 124 NaCl, 5 KCl, 1.25 NaH_2_PO_4_, 1.2 MgSO_4_, 26 NaHCO_3_, 2 CaCl_2_, and 10 glucose, in 30 mins before the start of the fast voltage-sensitive dye (Di-4-ANEPPS, Biotium, CA) staining procedure. Cells were stained with 0.2 mM Di-4-ANEPPS for 30 min in the incubation chamber and then transferred into the recording chamber. Imaging was performed at room temperature. Excitation light emitted by a shuttered green LED (LEX2, Brainvision, Tokyo, Japan) was reflected toward the cells through an excitation filter (531 nm wavelength). Emitted fluorescence signals passed through an absorption filter (580 nm wavelength) was imaged (0.5 ms frame rate; 6–8 min time lapse period) by a MiCam05 CMOS-based camera (SciMedia) with a Leica Plan APO 5x objective (NA: 0.5, Leica Microsystems, Wetzlar, Germany). The imaging data were acquired and analyzed using Brainvision Analysis Software (Brainvision). Fluorescence intensity changes (∆F/F) were normalized to baseline fluorescence recorded during the initial 10 ms of each recording and represented by pseudo-colors. Red color indicated a membrane depolarization, while blue color indicated a membrane hyperpolarization.

### Preparation of fibrils

Human α-Syn monomer protein for making pre-formed fibrils (PFFs) (1 mg aliquots, Proteos, cat. RP003) were generated over a seven-day period. Aliquots were thawed on ice for approximately 3 h. Once thawed, aliquots were centrifuged at 4 °C and 14.8 RPM. The supernatant was obtained and transferred into an autoclaved 1.5 mL microcentrifuge tube. Protein concentration of each aliquot was determined by a NanoDrop 2000 Spectrophotometer using the A280 protein method. 2 μL of 10x DPBS was used as a blank, followed by 2 μL of sample. Beer’s law was used to measure concentration (ε = 5960; kDa = 14.6). PFFs were diluted into 10x DPBS for a final concentration of 5 mg/mL. Tubes were vortexed for 3 s and lids were locked to prevent opening during the shaking process. Tubes were placed in an Eppendorf Thermomixer R at 37 °C. PFFs were shaken for 7 days (168 h) at 1000 RPM. Following shaking period, PFFs were aliquoted into 25 μL samples using gel loading pipet tips. Aliquots were frozen on dry ice and stored at − 80 °C until use in experiments.

### Treatments

Cells were exposed to PFFs (25 μg/ml), TTX (1 μM)), Picrotoxin (100 μM) or KCL (10 mM) alone or in combination in order to modulate cell activity. α-Syn PFFs were sonicated in pulses at approximately 1 pulse/second for 60 s before use. In some experiments, cultures we pre-treated with TTX, Picrotoxin or KCL for one hour at 37 °C and 5% CO_2_, then PFFs for 3 days. Cells were also co-incubated with TTX, Picrotoxin, KCL and/or PFFs for 3 days. Each experiment was performed in triplicate.

### In vitro direct current stimulation (DCS)

DCS was delivered to cells in each well of a custom-built 24-well culture plate (see Fig. 4a-c) through two L-shaped Ag/AgCl electrodes (0.5 mm diameter, Sigma, Oakville, ON) that were submerged in culture medium and connected to a Grass s8800 stimulator with a constant-current stimulus isolation unit (Grass Instrument Co., USA). Before each use, electrodes were sterilized using 70% ethanol for 15–20 min and washed with sterile culture medium. The stimulation intensity was set to achieve 50 mV/mm electric field for 40 mins and was applied following the 3-day incubation with PFFs. Immunohistochemistry, cell viability, and ELISA evaluation were performed 24 h after the DCS.

### Alpha-Synuclein ELISA

To measure the amount of α-Syn found extracellularly in cell culture medium, a sandwich ELISA kit (AnaSpec, Fremont, CA) was used. Following treatments, 300 μL per well of whole cell media was collected in duplicates from SH-SY5Y cells and stored at -80 °C until use. Samples were thawed, vortexed, and 100 μL of each diluted sample and standard was applied to microtiter plates pre-coated with anti-α-Synuclein monoclonal antibodies. Then, a detection antibody (Rabbit Polyclonal anti-α-Synuclein IgG-HRP, 10 μg/50 μl) was applied. After a four-hour incubation, wells were aspirated and washed with 350 μl/well of 1x wash buffer 6 times. Each wash included a 10 s lag time and was dried by inverting the plate and hitting it until no moisture appeared. The substrate was added and incubated at room temperature until a blue gradient was clearly observed (approximately 10 min). The colour reaction was measured using a Molecular Devices SpectraMax microplate reader at 450 nm within 20 min of adding the stop solution. The software (Soft-Max Pro) was used to create a standard curve and calculate the concentration of α-Syn in the samples.

### Evaluation of cell viability

To ensure the toxins and DCS were modulating cell activity without causing substantial cell death, a cytotoxicity assay was used. Cells that were incubated with fibrils alone or in combination with TTX, Picrotoxin or KCL for 3 days as well as DCS treated cells were analyzed. After incubation, cells had all media removed and replaced with complete media containing 2 drops/mL Hoeschst33342 (NucBlue™ live cell stain) and SYTOX (NucGreen™ dead cell indicator) (Invitrogen, Carlsbad, CA, USA). The plate was incubated at 37 °C with 5% CO_2_ for 15 min then imaged using the EVOS FL Imaging System (Invitrogen, Carlsbad, CA, USA). The counted nuclei of both dead (green) and live (blue) cells were averaged for six replicates and presented as a percentage of dead/live cells.

### Immunohistochemistry

Cells were fixed with 4% PFA in PBS for 15 min, then washed 3 × 5 minutes in PBS. Next, the cells were blocked with 2% BSA + 0.1% Triton-X in PBS for 30 min. The cells were subsequently incubated with anti-alpha synuclein filament antibody (1:1000, Abcam, Cambridge, MA, USA) which is a conformation specific antibody and specifically detects alpha-synuclein filaments or an anti-GABA_A_ receptor alpha 1 (Abcam, Cambridge, MA, USA) primary antibody at 1:250 in 0.1% BSA in PBS. Cells were washed 3 × 5 minutes in PBS, after which they were labelled with Alexa 488 anti-rabbit antibodies (Invitrogen, Carlsbad, CA, USA) at 1:1000 for 30 min at room temperature in 0.1% BSA in PBS. Cells were washed 3 × 5 minutes in PBS then imaged using the EVOS FL Imaging System (Invitrogen, Carlsbad, CA, USA).

### Fibril formation

Fibril formation was analyzed as total intensity using Image J (NIH). 30 cells were randomly selected per experimental condition, and total intensity was measured based on cell area and background. All evaluation and analysis were performed blindly.

### Data analysis and statistics

All experimental data are presented as mean ± standard error (S.E.M). Data were first analyzed for normality using the Shapiro-Wilk test and for equal variance using Levene’s method. The two-tailed unpaired or paired t test was performed for two-group comparisons. A one-way ANOVA followed by Tukey’s HSD post hoc tests was used for multi-group comparisons. Statistical significance was considered at *p* < 0.05.

## Results

### Preformed fibrils (PFF) treatment induced α-Syn inclusion accumulation in differentiated SH-SY5Y cells

**Fig. 1:**
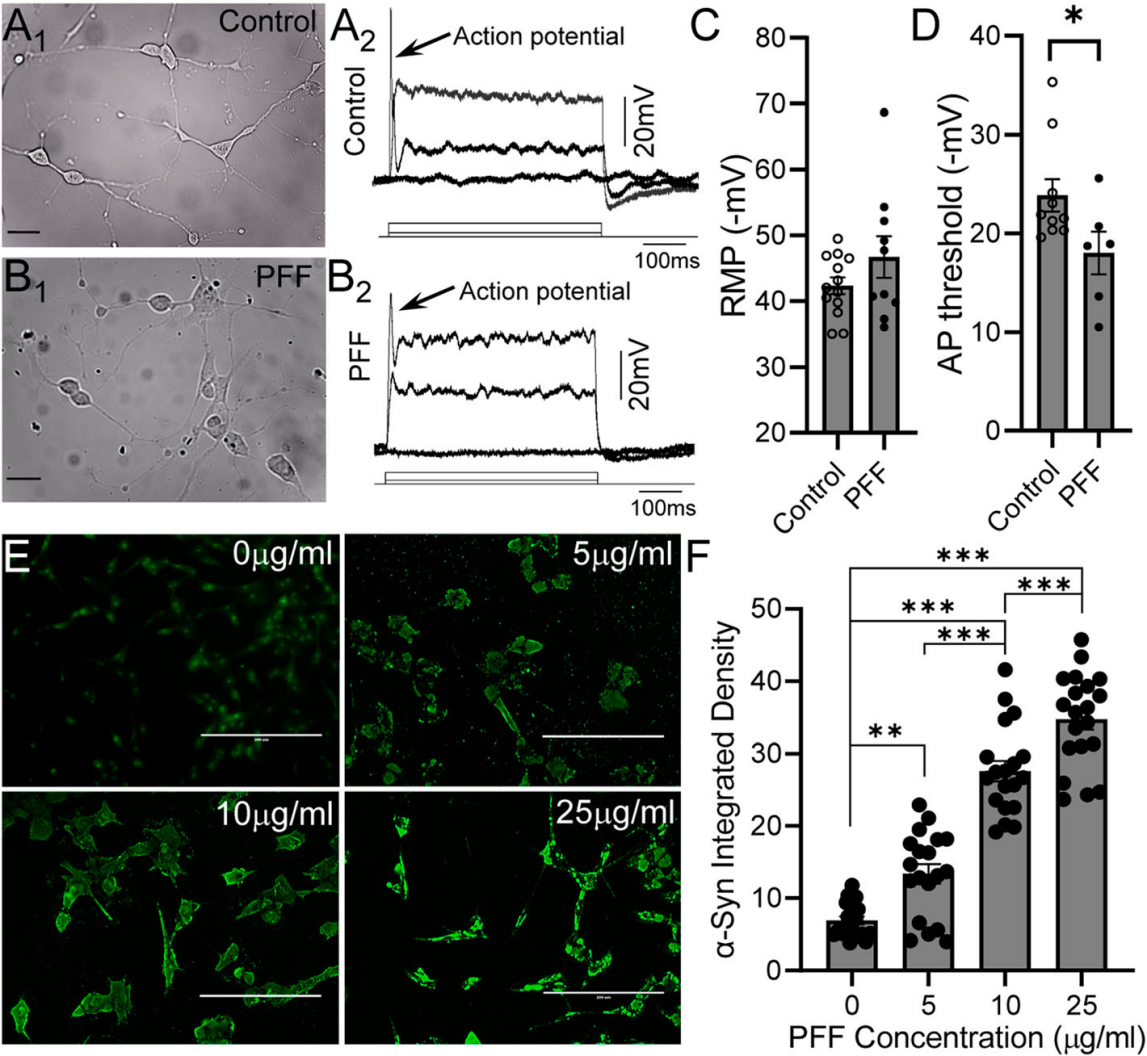
Preformed fibrils (PFFs) treatment induced α-Syn inclusion accumulations in the differentiated SH-SY5Y cells. **a**_**1**_ and **b**_**1**_ Representative IR-DIC image of differentiated SH-SY5Y neuroblastoma cells treated with (B_1_) or without (A_1_) 25 μg/ml PFFs treatment for 3 days. Note differentiated cells show long neurites and complex network connections. **a**_**2**_ and **b**_**2**_ Representative whole-cell voltage responses to 500 ms rectangular current injections of 0, 50, 120pA under the current clamp mode in differentiated SH-SY5Y cells treated with (B_2_) or without (A_2_) 25 μg/ml PFFs for 3 days. Arrow points to the action potentials generated. **c** Summary of the mean resting membrane potentials in control and 25 μg/ml PFFs-treated SH-SY5Y cells. Error bars indicate SEM. **d** Summary of the mean membrane potential threshold in control and 25 μg/ml PFFs-treated SH-SY5Y cells. Error bars indicate SEM. Two-tailed unpaired t test, *p < 0.05. **e** Immunofluorescent staining of α-Syn (green) in the differentiated SH-SY5Y cells following the treatment of 0, 5, 10, 25 μg/ml PFFs for 3 days. Scale bar: 200 μm. **f** Summary of the mean integrated intensity per cell of α-Syn staining in the differentiated SH-SY5Y cells following the treatment of 0, 5, 10, 25 μg/ml PFFs for 3 days. Error bars indicate SEM. One-way ANOVA, Tukey’s HSD post-hoc test. **p < 0.01. ***p < 0.001

In order to test the effects of membrane depolarization on α-Syn accumulation, we first added 0.1% RA to the culture medium for two weeks to differentiate the SH-SY5Y neuroblastoma cells. Consistent with previous studies [16, 22, 47], differentiated SH-SY5Y cells showed a variety of neuron-like phenotypes including long neurites and complex network connections (Fig. 1a_1_). Furthermore, whole-cell current clamp recordings demonstrated that while no spontaneous action potentials were detected, appropriate depolarizing current injections could evoke overshooting action potentials in differentiated SH-SY5Y cells (Fig. 1a_2_). We next proceeded to treat the differentiated SH-SY5Y cells with different concentrations (5, 10, and 25 μg/ml) of PFFs or vehicle for 3 days, and subsequently, evaluated α-Syn accumulation (Fig. 1e and f). The PFF treatments induced significant increases in α-Syn accumulation (5 μg/ml: 13.36 ± 5.89, *n* = 19, *p* < 0.001; 10 μg/ml: 27.58 ± 6.15, n = 19, p < 0.001; 25 μg/ml: 34.70 ± 6.47, *n* = 20, p < 0.001) compared to control cells (6.92 ± 2.44, n = 20). α-Syn accumulation appeared to increase with PFF concentrations and peaked in 25 μg/ml PFF-treated SH-SY5Y cells (25 μg/ml vs 5 μg/ml: p < 0.001; 25 μg/ml vs 10 μg/ml, p < 0.001). In addition, PFFs (25 μg/ml) did not cause a significant change in resting membrane potentials (RMPs), but there was a trend towards hyperpolarization (− 46.7 ± 3.2 mV, *n* = 10, *p* = 0.18) compared to control SH-SY5Y cells (− 42.3 ± 1.3 mV, *n* = 13, Fig. 1c. This was associated with an increase in the action potential threshold in PFF-treated SH-SY5Y cells (PFF: 18.0 ± 2.2 mV, *n* = 6 vs Control: − 23.9 ± 1.6 mV, n = 10, *p* = 0.049, Fig. 1d). These results suggest that 3-day PFF treatment induced significant pathological α-Syn accumulation in differentiated SH-SY5Y cells.

### Membrane depolarization significantly reduces α-Synuclein fibril accumulation in differentiated SH-SY5Y cells

**Fig. 2:**
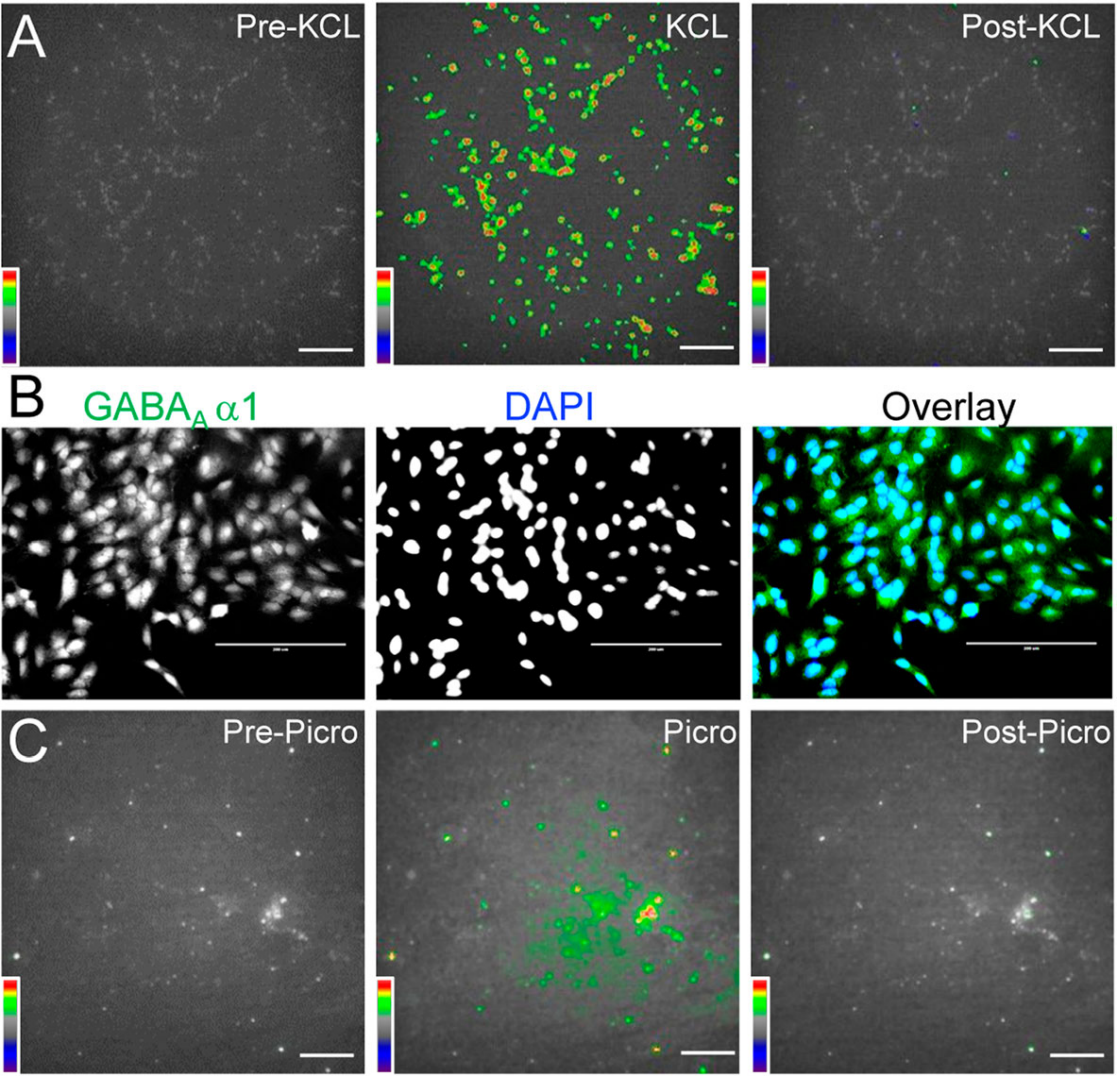
Membrane depolarization induced by increasing extracellular K^+^ and blocking GABA_A_ receptors in differentiated SH-SY5Y cells. **a** Representative responses (∆F/F) of Fast voltage-sensitive dye imaging of Di-4-ANEPPS-loaded SH-SY5Y cells before, during, and after 10 mM KCL. Scale bar, 100 μm. **b** Immunofluorescent staining of GABA_A_ α1(green) and DAPI (blue) in the differentiated SH-SY5Y cells. Scale bar: 200 μm. **c** Representative responses of Fast voltage-sensitive dye imaging of Di-4-ANEPPS-loaded SH-SY5Y cells before, during, and after 100 μM Picrotoxin. Scale bar, 100 μm

We next sought to assess the effects of membrane depolarization on α-Syn fibril accumulation in differentiated SH-SY5Y cells**.** To achieve membrane depolarization, we employed two independent strategies rather than simply one in order to avoid possible stimulation-specific effects. 1) By increasing extracellular K^+^ concentration in the culture medium through addition of 10 mM KCL. Fast voltage-sensitive dye imaging of Di-4-ANEPPS-loaded SH-SY5Y cells confirmed high extracellular K^+^-induced membrane depolarization (Fig. 2a, *n* = 5). 2) Blockade of the inhibitory GABA_A_ receptors by specific inhibitor Picrotoxin (100 μM) was applied to depolarize the cell membrane. Immunohistochemistry with an antibody against GABA_A_ receptor α1subunit confirmed the expression levels of GABA_A_ receptors in differentiated SH-SY5Y cell**s (**Fig. 2b, *n* = 4**)**. Consistent with the expression of GABA_A_ receptors, fast voltage-sensitive dye imaging showed GABA_A_ receptor inhibitor Picrotoxin evoked membrane depolarization in differentiated SH-SY5Y cell**s** (Fig. 2c, n = 4). These results provide functional evidence of KCL and Picrotoxin induced membrane depolarization in differentiated SH-SY5Y cell**s.**

**Fig. 3:**
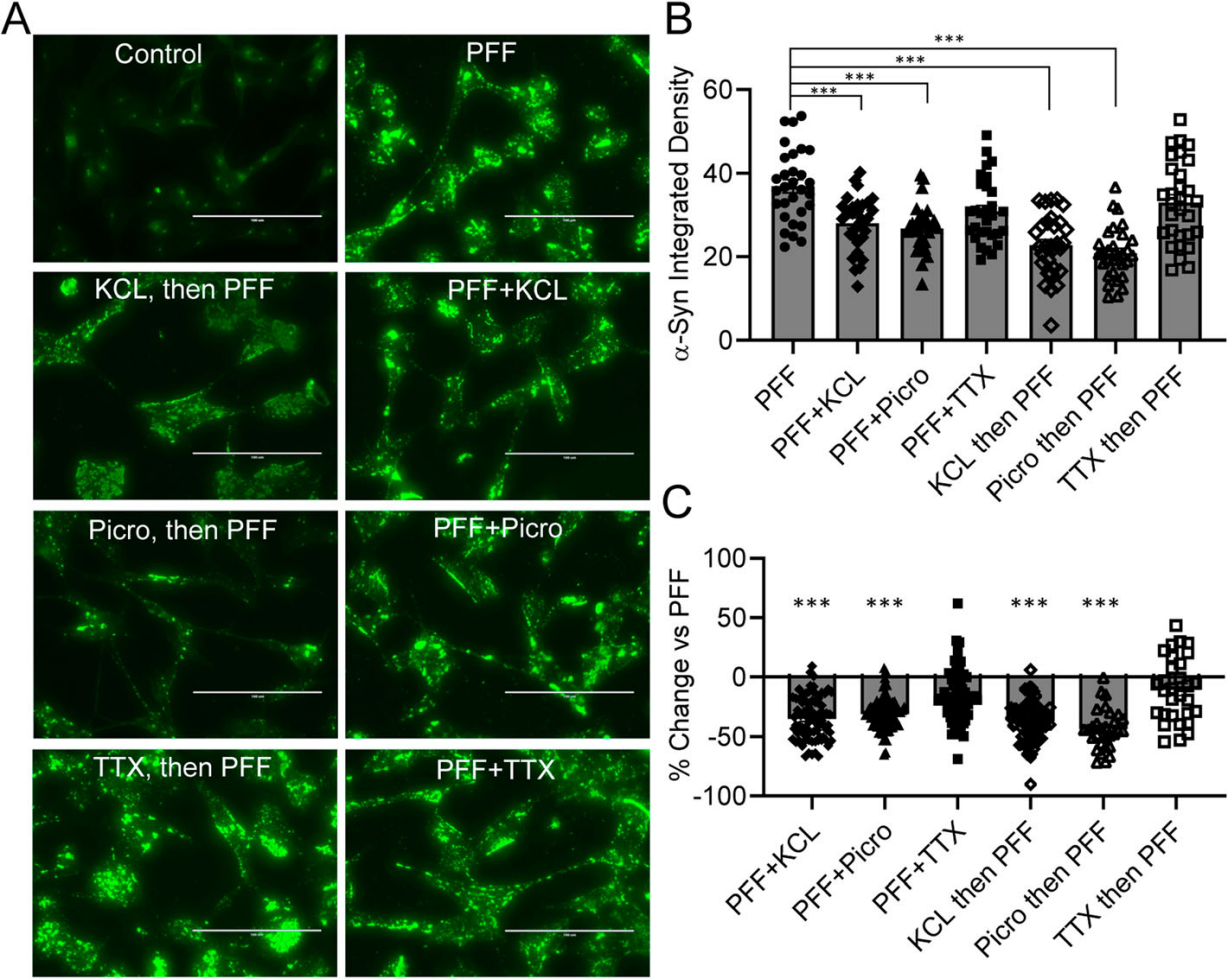
Membrane depolarization induced by KCL and Picrotoxin reduces α-Syn aggregation in PFF-treated SH-SY5Y cells. **a** Immunofluorescent staining of α-Syn (green) in the differentiated SH-SY5Y cells following the treatment of control, PFF only, KCL then PFF, Picrotoxin then PFF, TTX then PFF, PFF + KCL, PFF + Picrotoxin, or PFF + TTX for 3 days. Scale bar: 200 μm. **b** Summary of the mean integrated intensity per cell of α-Syn staining in the differentiated SH-SY5Y cells following the treatment of PFF only, KCL then PFF, Picrotoxin then PFF, TTX then PFF, PFF + KCL, PFF + Picrotoxin, or PFF + TTX for 3 days. Error bars indicate SEM. One-way ANOVA, Tukey’s HSD post-hoc test. ***p < 0.001. **c** Average percentage changes expressed as percentage of the PFF only group in the integrated intensity per cell of α-Syn staining in the differentiated SH-SY5Y cells following the treatment of KCL then PFF, Picrotoxin then PFF, TTX then PFF, PFF + KCL, PFF + Picrotoxin, or PFF + TTX for 3 days. Error bars indicate SEM. One-way ANOVA, Tukey’s HSD post-hoc test. ***p < 0.001 compared with the PFF only group

We then examined whether these treatments affect α-Syn fibril accumulation in differentiated SH-SY5Y cell**s.** The effects were evaluated by quantifying the integrated density of α-Syn fibril immunostaining. After 72 h of treatment with 100 μM Picrotoxin or 10 mM KCL in combination with 25 μg/ml PFFs, the accumulation of α-Syn fibrils in differentiated SH-SY5Y cells significantly decreased by 30.98% (PFF + Picro: Integrated Intensity 26.77 ± 1.03, *n* = 30, *p* < 0.0001) and 34.96% (PFF + KCL: Integrated Intensity 28.03 ± 1.21, n = 30, p < 0.0001), compared to the PFF-only group (Integrated Intensity 36.92 ± 1.58, n = 30, Fig. 3a-c). Furthermore, after 1 h pre-treatment with 100 μM Picrotoxin or 10 mM KCL followed by 72 h treatment with 25 μg/ml PFFs, the accumulation of α-Syn fibrils in differentiated SH-SY5Y cells also significantly decreased by 43.61% (Picro then PFF: Integrated Intensity 20.82 ± 1.14, n = 30, p < 0.0001) and 36.85% (KCL then PFF: Integrated Intensity 22.74 ± 1.35, n = 30, p < 0.0001), respectively, compared to the PFF-only group (Fig. 3a-c). In addition, there was a significant difference in the integrated density of α-Syn fibril immunostaining between the group that received combined PFF + Picro, compared to those that first received Picrotoxin followed by PFFs (*p* = 0.0208); however, no difference was evident between the combined PFF + KCL, compared to those administered KCl and then PFFs (*p* = 0.9878). Interestingly, consistent with the fact that no spontaneous action potentials were detected in differentiated SH-SY5Y cells, the sodium channel blocker TTX (1 μM) in combination with 25 μg/ml PFFs or 1 h pre-treatment with TTX did not induce significant changes in α-Syn fibril accumulation (PFF + TTX: Integrated Intensity 30.85 ± 1.42, n = 30, *p* = 0.0692; TTX then PFF: Integrated Intensity 33.02 ± 1.81, n = 30, *p* = 0.4521), compared to compared to the PFF-only group (Fig. 3a-c). Collectively, these data strongly suggest that membrane depolarization is crucial for eliminating α-Syn fibril accumulation in PFF-treated differentiated SH-SY5Y cells.

### Direct current stimulation (DCS) significantly alleviates α-Synuclein fibril accumulation in differentiated SH-SY5Y cells

**Fig. 4:**
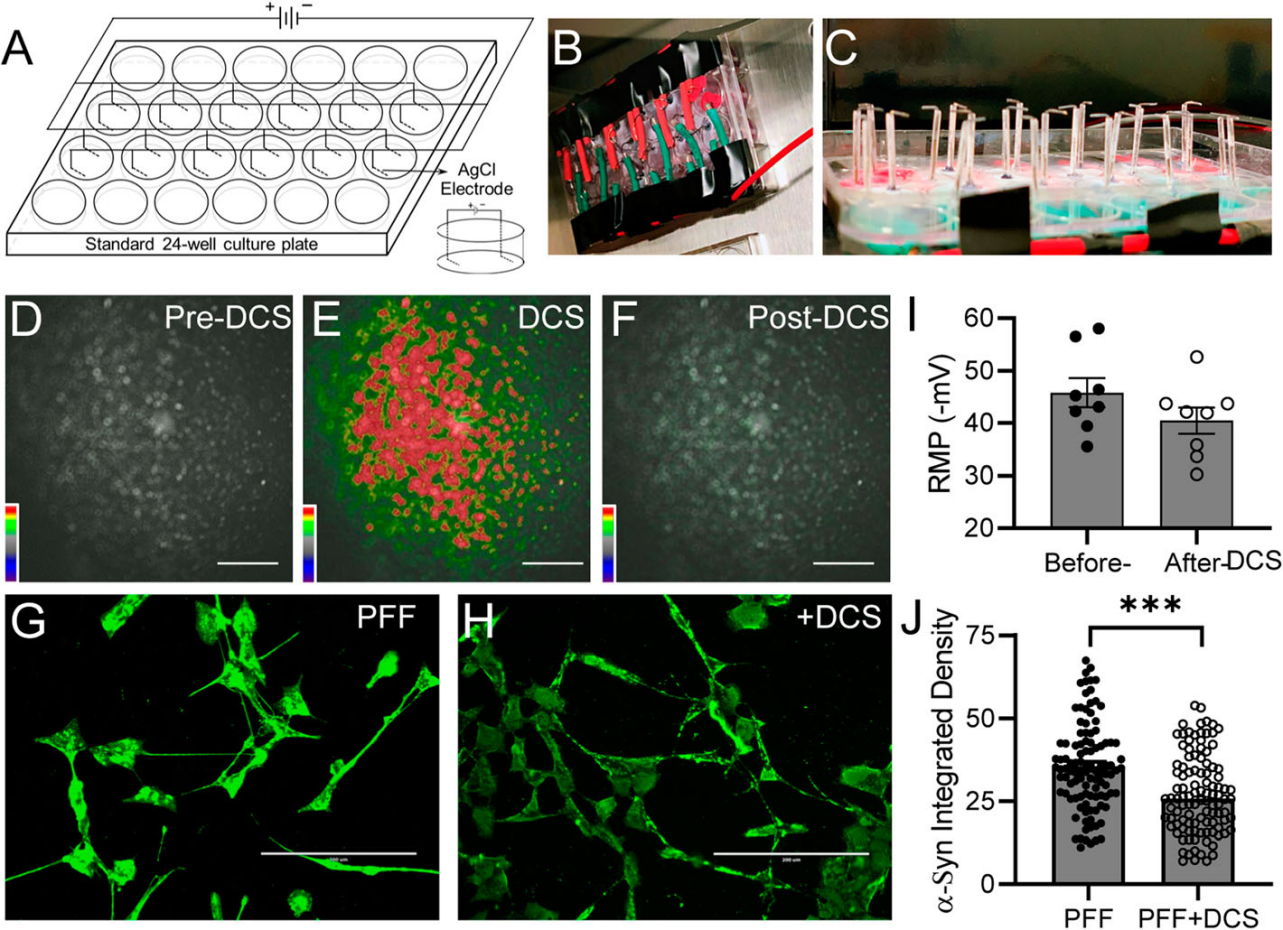
Direct current stimulation (DCS) alleviates α-Syn aggregation in PFF-treated SH-SY5Y cells. **a-c** Custom-built 24-well culture plate for DCS. Note each well has a pair of L-shape Ag/AgCl electrodes connected to the stimulator. **d-f** Representative response (∆F/F) of differentiated SH-SY5Y cells to before (**d**), during (**e**) and after (**f**) 50 mV/mm DCS stimulation during voltage sensitive dye imaging. Scale bar, 100 μm. **g-h** Immunofluorescent staining of α-Syn (green) in the differentiated SH-SY5Y cells with (right panel) or without (left panel) 50 mV/mm DCS stimulation for 30mins. Scale bar: 200 μm. **i** Summary of the mean resting membrane potentials in the PFF-treated differentiated SH-SY5Y cells before or after 50 mV/mm DCS stimulation. Error bars indicate SEM. **j** Summary of the mean integrated intensity per cell of α-Syn staining in the PFF-treated differentiated SH-SY5Y cells with or without 50 mV/mm DCS stimulation. Error bars indicate SEM. Two-tailed unpaired t test. ***p < 0.001

Given the promising protective effects of KCL/Picrotoxin-induced membrane depolarization in clearing α-Syn fibril accumulation, we further evaluated whether the clinically relevant DCS treatment would induce a similar outcome. To this end, PFF-treated differentiated SH-SY5Y cells were exposed to 0 or 50 mV/mm DCS with constant frequency of 50-100 Hz for 40 mins through L-shape Ag/AgCl electrodes in a custom-built 24-well culture plate (Fig. 4a-c). Using optical fast voltage-sensitive dye imaging, we confirmed that 50 mV/mm DCS evoked robust membrane depolarization in differentiated SH-SY5Y cells as evidenced by robust red fluorescent signals (Fig. 4d-f, *n* = 4). After 40mins DCS treatment in combination with 25 μg/ml PFFs, the accumulation of α-Syn fibrils in differentiated SH-SY5Y cells significantly reduced by 23.26% (Integrated Intensity 27.28 ± 1.16, *n* = 106, *p* < 0.0001, compared to the PFF-only group (Integrated Intensity 35.55 ± 1.28, n = 106, Fig. 4g-h and j). Importantly however, the stimulation intensity of 50 mV/mm did not cause detectable changes in cell morphology or significant long-lasting membrane depolarization after DCS treatment (RMP: before DCS -45.8 ± 2.8 mV, *n* = 8 vs after DCS -40.5 ± 2.5 mV, n = 8, *p* = 0.17, Fig. 4i). These results demonstrated a protective effect of DCS on the clearance of α-Syn fibril accumulation in differentiated SH-SY5Y cells.

### Membrane depolarization causes the secretion of α-Synuclein fibrils into the extracellular medium in differentiated SH-SY5Y cells

**Fig. 5:**
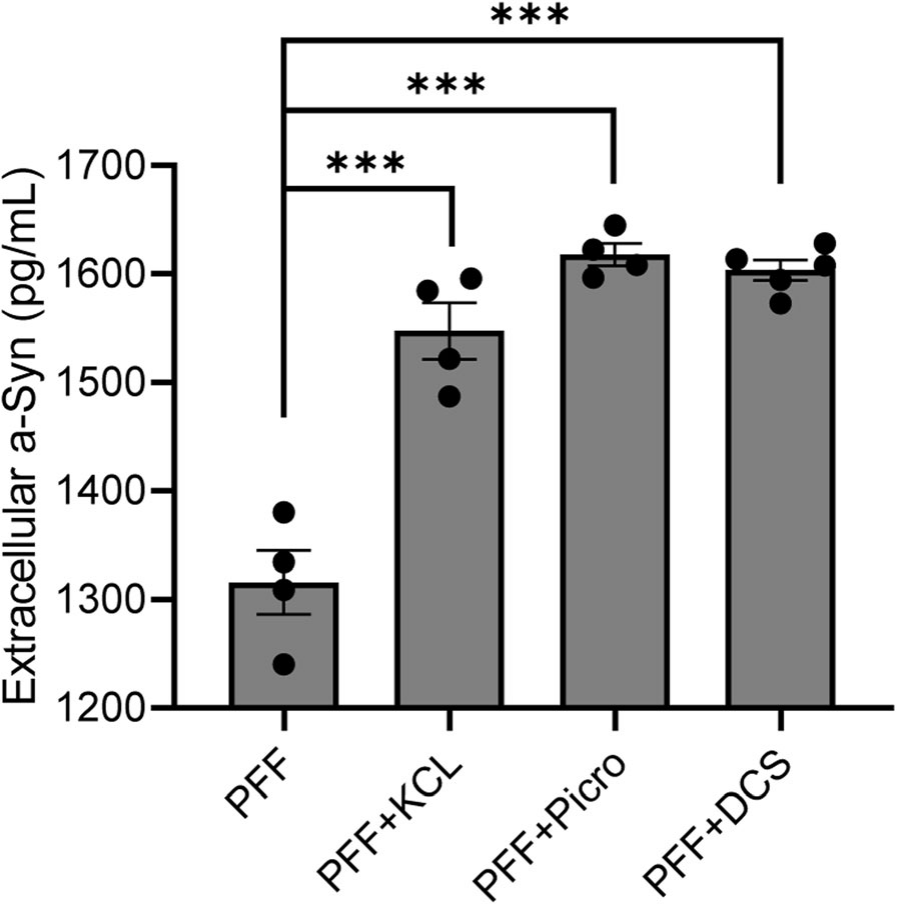
Membrane depolarization causes the secretion of α-Syn into the extracellular medium. The levels of α-Syn in medium as measured by ELISA were increased following 10 mM KCL, 100 μM PTX or DCS treatment compared to the PFF alone group. Error bars indicate SEM. One-way ANOVA, Tukey’s HSD post-hoc test. ***p < 0.0001. Medium obtained from cultured SH-SY5Y cells

To investigate the mechanism of membrane depolarization induced α-Syn fibril clearance, an ELISA was used determine the total amount of α-Syn in the extracellular medium following membrane depolarization. To this end, the concentration of extracellular total α-Syn in the culture medium was significantly elevated following membrane depolarization evoked by KCL (1547 ± 25.72 pg/ml, n = 4, p < 0.0001), PTX (1618 ± 10.38 pg/ml, n = 4, p < 0.0001) or DCS (1603 ± 9.341 pg/ml, *n* = 5, p < 0.0001) compared to PFF alone (1316 ± 29.27 pg/ml, n = 4, Fig. 5). Importantly, this coincides with the decreases in intracellular α-Syn fibril accumulation following membrane depolarization (as demonstrated by immunohistochemistry). These results strongly support the notion that membrane depolarization alleviates intracellular α-Syn aggregation, at least in part through promoting α-Syn secretion.

### Membrane depolarization does not cause cell death in PFF-treated differentiated SH-SY5Y cells

**Fig. 6:**
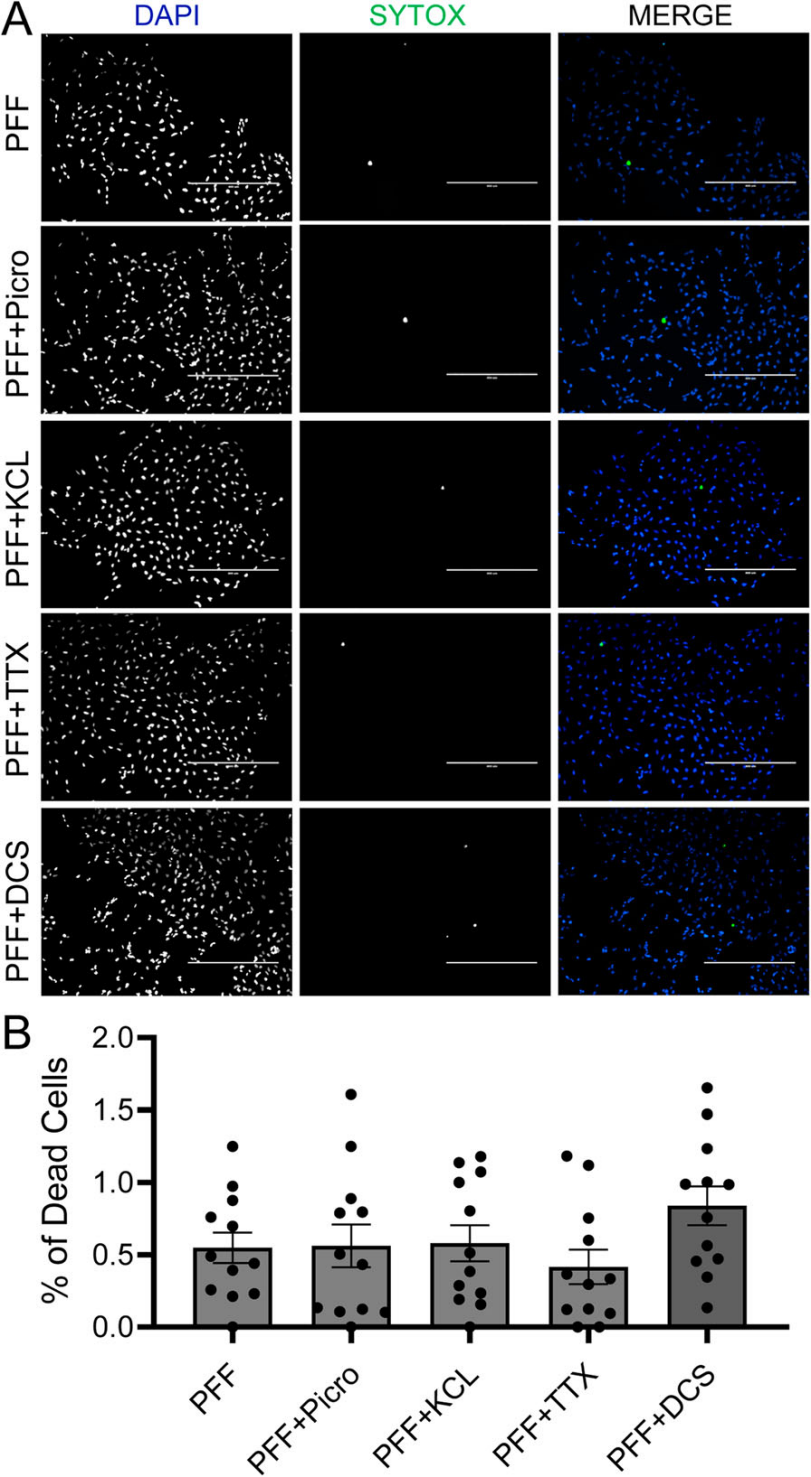
KCL, Picrotoxin, TTX and DCS treatments did not cause cell death in PFF-treated SH-SY5Y cells. **a** Representative NucGreen™ staining (SYTOX, green; DAPI, blue) of PFF-treated differentiated SH-SY5Y cells treated with 10 mM KCL, 100 μM Picrotoxin, 1 μM TTX, and DCS. Scale bar: 200 μm. **b** Quantification of average live/dead ratio of PFF-treated differentiated SH-SY5Y cells with 10 mM KCL, 100 μM Picrotoxin, 1 μM TTX, or DCS treatments. Error bars indicate SEM. One-way ANOVA, Tukey’s HSD post-hoc test

We next examined whether membrane depolarization-induced elimination of α-Syn accumulation resulted from any changes in cell death. Cytotoxicity was determined by the live/dead ratio calculated through NucGreen™ staining. In PFF only treated (25 μg/ml) differentiated SH-SY5Y cells, the distribution of NucGreen positive cells was very sparse (0.55 ± 0.11%, *n* = 12). Further, membrane depolarization induced by administration of additional treatment of 10 mM KCL (0.58 ± 0.43%, n = 12, *p* = 0.99), 100 μM Picrotoxin (0.56 ± 0.51%, n = 12; p = 0.99), 1 μM TTX (0.42 ± 0.41%, n = 12; *p* = 0.95), or DCS (0.84 ± 0.46%, n = 12, *p* = 0.50) did not cause significant changes in cytotoxicity compared to the background level seen with PFF only treatment (Fig. 6a and b). Collectively, these results suggest that membrane depolarization-induced changes in α-Syn accumulation were not mediated by treatment induced increases in cytotoxicity in differentiated SH-SY5Y cells.

## Discussion

Widespread toxic α-Syn inclusions are a hallmark of PD pathology [18, 19, 21, 38] and novel methods of inducing clearance of α-Syn aggregation may be a promising potential therapeutic approach [23, 38, 51]. In the present study, using an in vitro α-Syn PFF model of PD in human neural cells, we demonstrated for the first time that membrane depolarization plays an important role in alleviating PFF-induced toxic α-Syn aggregates. We showed that PFF treatment for 3 days reliably induced a robust dose-dependent α-Syn accumulation in differentiated human neural cells. Importantly, functional membrane depolarization induced by either high extracellular K^+^ or the GABA_A_ receptor blocker significantly alleviated the toxic α-Syn aggregation. We further demonstrated that clinically relevant DCS also remarkably decreased toxic α-Syn aggregation in PFF-treated human neural cells. Furthermore, this membrane depolarization-induced decrease in intracellular α-Syn observed with KCL, PTX and DCS was coupled with an increase in extracellular α-Syn. Importantly, the treatments used in the present study did not induce confounding cell cytotoxicity. Our results provide direct evidence of an important interplay between membrane depolarization and clearance of intracellular α-Syn aggregation.

α-Syn PFFs have been repeatedly shown to initiate Lewy body pathological changes through templating and recruiting endogenous α-Syn to accumulate and form insoluble inclusions in neurons in a variety of PD models [9, 14, 24, 26, 36, 47]. Here, we used an α-Syn PFF model in the differentiated SH-SY5Y cells which have already been extensively used in PD research due to their human-specific proteins [3], dopaminergic phenotypes [1, 3, 41], and neuronal electrophysiological properties, i.e., overshooting action potentials [48] (Fig. 1b). Consistent with previous studies [9, 47], our results showed that α-Syn PFF treatment effectively induced endogenous α-Syn inclusions and modulated membrane properties in the differentiated SH-SY5Y cells. Prolonged exogenous PFF treatment for two weeks has previously been reported to lead to neurotoxicity in primary hippocampal neurons [50]. In the present study, to avoid the sustained PFF treatment-induced cell death, a higher concentration (25 μg/ml) of α-Syn PFF was applied for a relatively short period of time (3 days). Under these conditions, toxic α-Syn inclusions were induced with no detectable cell toxicity in either PFF only or PFF in combination with KCL/TTX/Picrotoxin/DCS treated SH-SY5Y cells (Fig. 6), supporting that changes in the levels of α-Syn inclusions were not due to treatment-induced cell death.

An important finding of our study is that functional membrane depolarization has a crucial role in clearing PFF-induced toxic α-Syn aggregation. Previous in vivo and in vitro studies have shown that depolarization or increased neuronal activity can increase the mobility of physiological α-Syn monomers and promote their disassociation from presynaptic membranes and release to extracellular spaces through a Ca^2+^ dependent mechanism [12, 13, 17, 52]. In undifferentiated human cell lines overexpressing physiological α-Syn monomers, however, a transit (about an hour) elevation of intracellular Ca^2+^ through the addition of high extracellular KCL or Ca^2+^ levels can induce an increase in intracellular α-Syn aggregation [15, 34, 40], while this same treatment also promoted the extracellular secretion of α-Syn monomers after longer elevations (6 h) of intracellular Ca^2+^ in differentiated human neuroblastoma cells [12]. These studies support a potential multifactorial interplay between the intracellular calcium levels and α-Syn dynamics. It is important to note that the high level of α-Syn monomers used in these studies can increase the potential mitochondrial oxidative stress [10] and the vulnerability of α-Syn aggregation [18, 27] and thus may introduce confounds while evaluating the α-Syn toxicity. Currently, it is still not entirely clear whether membrane depolarization can affect endogenous α-Syn fibrilization during pathological conditions.

In the present study, we used human wild-type PFF-treated human neural cells to seed endogenous α-Syn inclusions without the additional complications of α-Syn overexpression. Two independent pharmacological strategies were used to evoke functional membrane depolarization to avoid the possibility of stimulation-specific effects. We revealed that membrane depolarization evoked by either KCL or PTX for 3 days alleviated the PFF-induced toxic α-Syn aggregates in human neural cells by significantly increasing the secretion of α-Syn into the extracellular media (Figs. 3 and 5). Whether or not the secreted α-Syn has undergone conformational changes will require further investigation. Furthermore, while we found that chronic membrane depolarization for 3 days did not induce detectable cytotoxicity, we cannot exclude the potential possibility of excitotoxicity due to prolonged Ca^2+^ influx. The time course of depolarization treatment may need to be further optimized to balance any potential excitotoxicity with alterations in the clearance of α-Syn aggregates. Membrane depolarization might enhance the clearance of the toxic α-Syn aggregates by upregulating the expression of heat shock protein 70 (HSP70) and the activation of autophagy, both of which have been shown to promote the clearance of α-Syn inclusions [18, 20, 29, 53]. Interestingly, TTX did not cause a significant change in the amount of α-Syn accumulation regardless of when it was administered. The lack of response seen with TTX is in line with the perspective that SH-SY5Y cells do not show spontaneous action potentials during the resting condition. It is of interest that PFFs have been shown to impair the initiation of synaptogenesis and synaptic function in cultured excitatory hippocampal neurons [51]. This might, in turn, induce further α-Syn fibrilization and synaptic dysfunction resulting in a vicious cycle to promoting toxic α-Syn aggregates in synucleinopathies. Further research is needed to explore the molecular mechanisms mediating the membrane depolarization-induced disaggregation. Although we can not exclude the possibility that our alpha Synuclein filament antibody may label PFFs in addition to endogenous alpha-Syn fibrils, our results clearly demonstrate that membrane depolarization does significantly decrease the levels of intracellular alpha-Syn fibrils (PFF or endogenous alpha-syn fibrils), and importantly, also increased the extracellular secretion of a-Syn. Therefore, membrane depolorization might represent an effective strategy to interfere with the intitiation of synaptic dysfunction-α-Syn fibrilization in PD pathophysiology.

Finally, we induced membrane depolarization by applying an external electrical field using a clinically relevant DCS procedure. Physiological α-Syn contains two structural domains, the N-terminal lipid-binding domain including a hydrophobic NAC (non-amyloid-β component) and the C-terminal domain [4, 28, 31]. Toxic α-Syn fibril formation requires significant α-Syn conformational changes from α-helix to β-sheet to expose hydrophobic NAC, leading to the formation of α-Syn protofibrils and fibrils in neurons [7, 45]. Interestingly, the α-Syn N-terminal is positively charged, while the C-terminal is negatively charged [11, 31, 49]. Therefore, theoretically, an external electrical field could disrupt α-Syn aggregation by aligning α-Syn with induced dipoles following the axis of the electric field or directly interacting with the α-Syn dipoles to induce a β-sheet to α-helix conformational switch [2, 42]. Indeed, using a novel, custom-built 24-well culture plate, we obtained direct evidence for the first time to demonstrate that clinically relevant DCS effectively alleviated the accumulation of PFF-induced toxic α-Syn aggregates in differentiated human neural cells, very much the same as we observed with high extracellular K^+^ and the GABA_A_ receptor blocker picrotoxin. Interestingly, Electroconvulsive therapy (ECT) has been shown to cause antiparkinsonian effects in patients with advanced PD [32, 33]. Furthermore, we have recently shown that epilepsy was associated with significant improvement in clinical motor symptoms and cognitive decline in PD patients [39]. While the mechanism behind these positive effects remains elusive, our findings suggest this effect may be due in part to the clearing of toxic α-Syn aggregates from dopaminergic neurons and offers beneficial information that could provide therapeutic benefits in controlling PD pathology. Future in vivo studies may wish to investigate the mechanism responsible for the interplay between membrane depolarization and α-Syn fibril clearance and the relationship to clinical improvement.

## Conclusion

Overall, employing several novel approaches that utilize a new in vitro α-Syn PFF model of PD, we have shown that membrane depolarization, through provocation of α-Syn fibril secretion, can protect against intracellular accumulation of toxic PFF-induced α-Syn aggregates and importantly, that this occurs in the absence of any cell death. The present data could have important clinical implications for synucleinopathies, specifically PD, and may also help determine a mechanism of action for the accumulation of α-Syn aggregates.

## Data Availability

The datasets used and/or analyzed during the current study available from the corresponding author on reasonable request.

## Abbreviations

PD: Parkinson’s disease
α-Syn: alpha-synuclein
PFF: Preformed fibril
DCS: Direct current stimulation
DMEM: Dulbecco’s Modified Eagle Media
FBS: Fetal bovine serum
PDL: Poly-D-Lysine
LM: Laminin
RA: Retinoic acid
ACSF: Artificial cerebral spinal fluid
TTX: Tetrodotoxin
KCL: Potassium chloride
PFA: Paraformaldehyde
PBS: Phosphate-buffered saline
BSA: Bovine serum albumin
GABA_A_: γ-Aminobutyric acid type A
HSP70: Heat shock protein 70
NAC: Non-amyloid-β component
ECT: Electroconvulsive therapy
